# Dataset on field estimation of vegetation cover loss due to charcoal production in Afram Plains of Ghana

**DOI:** 10.1016/j.dib.2024.111022

**Published:** 2024-10-26

**Authors:** Thelma Arko

**Affiliations:** Utrecht University, the Netherlands

**Keywords:** Deforestation, Data collection, Charcoal production, Environmental impact, Sustainable cooking fuel

## Abstract

The impact of urban charcoal consumption on tree cover loss in Ghana remains understudied, with limited data and inconsistent methodologies hindering a comprehensive understanding. This data article addresses these gaps by presenting a valuable dataset on charcoal production and its environmental implications in the Afram Plains of Ghana. A systematic data collection process was undertaken, encompassing 29 charcoal production sites across four communities: Tease, Odumasua, Anlo Fasso, and Forifori. Semi-structured interviews with community elders, chiefs, and charcoal producers provided insights into the historical context and local knowledge of charcoal production activities.

The dataset includes a wealth of information, such as land use characteristics, the number of trees utilized for charcoal production, and measurements of tree stump diameters, lengths, and volumes. Local names and scientific identification of tree species were recorded, offering a detailed understanding of the vegetation impacted by charcoal production.

The potential for reuse of this dataset is significant. Researchers can utilize the information to further explore the complex dynamics between charcoal production and tree cover loss develop evidence-based policies, and promote sustainable alternatives. By making this dataset publicly available, we encourage its reuse to support interdisciplinary research, enhance understanding of charcoal production's environmental footprint, and inform decision-making processes aimed at preserving Ghanaʼs valuable vegetation cover.

Specifications Table

**Table:** 

Subject	Environmental Science
Specific subject area	Charcoal Production. This is a wood-based activity that involves in most cases indiscriminate cutting of trees, preferably hard wood species from the open forest and subjecting it to anaerobic combustion conditions to produce a black coal called “charcoal”. Charcoal is a very important heat source for cooking in most parts of sub-Saharan Africa.
Type of data	Raw
Data collection	Data was collected through field surveys at 29 charcoal production sites across four communities in the Afram Plains of Ghana. Tools used included a GPS fields area measure app for land area estimation, a handheld surveyor's tape for measuring tree stump lengths, and a flexible measuring tape for trunk diameters. Data was recorded using a mobile app synchronized with GIS cloud mapping software (Mobile Data Collection Portal). Semi-structured interviews with community elders, chiefs, and charcoal producers provided contextual information. Measurements included land use characteristics, tree counts, stump dimensions, species identification, and charcoal production metrics.
Data source location	The data was collected from Afram Plains located in the northern-most part of the Eastern Region in Ghana, on Latitude 6.912698° and Longitude −0.141224.
Data accessibility	**Please note:** All raw data referred to in this article must be made publicly available in a data repository prior to publication. Please indicate here where your data are hosted (the URL must be working at the time of submission and editors and reviewers must have anonymous access to the repository): Repository name: Mendeley data Data identification number: 10.17632/vv38xp886m.1 Direct URL to data: Tree Charcoal Data Afram Plains - Mendeley Data Hover to copy the Url or Cntrl+ Click and follow the link
Related research article	Arko, T., Mensah, A., Adomako, J., Denton, F., & Obani, P. (2024). The multifaceted socio-ecological impacts of charcoal production on the Afram Plains, Ghana. Trees, Forests and People, 16. 10.1016/j.tfp.2024.100586

## 1 Value of the Data

• These data are valuable as they shed light on the often overlooked issue of charcoal production and its impact on tree cover loss in Ghana.
• The data emphasizes the research gaps and the limited understanding of the actual impact of charcoal production activities on the environment. This calls attention to the importance of empirical research in this field, which can help policymakers and researchers to develop sustainable charcoal production practices and strategies.
• Other researchers can reuse these data to advocate for improved data collection methods and consistent methodologies specific to charcoal production. This includes highlighting the limitations of relying solely on extrapolations and projections and the need for up-to-date, accurate information to support policy initiatives aimed at sustainable charcoal management.
• The dataset provides a basis for further investigations into the links charcoal production and deforestation. By analyzing these data, researchers can explore the implications of charcoal production and consumption by the growing population without access to modern cooking fuel sources, contributing to a more comprehensive understanding of the environmental and social impacts of this industry.
• Additionally, the data can be utilized to assess the effectiveness of current policies and strategies related to wood fuel consumption. By comparing the available information with the actual impact on tree covers, researchers can identify gaps and shortcomings in the current approaches, leading to more informed policy recommendations and interventions.

## 2 Background

The motivation behind compiling this dataset stems from the recognized need to address the impact of urban charcoal consumption on tree cover loss in Ghana. Previous studies have highlighted the lack of reliable data and consistent methodologies to understand the complex relationship between charcoal production and deforestation. The theoretical background of this research is rooted in the field of environmental science, specifically exploring the sustainability of charcoal as a cooking fuel source within urban areas. The context is the growing urban population in Africa, increasing the demand for charcoal, and the subsequent pressure on rural landscapes, leading to deforestation.

This dataset adds value to the original research article by providing a detailed account of the data collection process, research design, and analytical procedures employed. It offers transparency and enables other researchers to replicate and build upon the work. Additionally, the dataset includes raw data and calculations, allowing for further analysis and exploration of the relationships between various attributes. The data article complements the original research [1] by providing a comprehensive understanding of the impact of charcoal production on vegetation cover and contributing to evidence-based policy-making.

## 3 Data Description

The dataset is contained in a single CSV file named “tree charcoal data thesis edited08august2023_published_mendeley.csv”. The file consists of 15 columns and 41 rows of data.

This study collected data from 29 charcoal production sites across four communities in the Afram Plains of Ghana: Tease (4 sites), Anlo fasso (4 sites), Odumasua (4 sites), and Forifori (17 sites). Each site represents a unique charcoal production area within these communities. The dataset contains information on various attributes for each site, including tree species, age, density, and charcoal production metrics

The columns are:

1. Site number
2. Community
3. Most abundant species
4. Median Age
5. No. of trees per Site
6. Stock Density/m^2^
7. No. of trees cut to make charcoal
8. Percentage of tree removal
9. Area of land (m^2^)
10. Area of land cleared for charcoal (m^2^)
11. No. of bags of charcoal produced
12. No of bags per tree
13. No. of trees per bag
14. Area of land per tree
15. Area of land per bag (m^2^)

The rows represent data collected from various sites across four communities: Forifori, Odumasua, Tease, and Anlofasso. Table 1 below shows a sample of the data:

**Table 1:** Sample data from charcoal production sites in the Afram Plains of Ghana.

Site number	Community	Most abundant species	Median Age	No. of trees per Site	...	Area of land per bag (m^2^)
AduanaSite 4	Tease	Pterocarpus sp Anogeissus sp	16	108	...	28.64
AduanaSite 1	Tease	Unknown	30	3	...	246.42
AduanaSite 2	Tease	Pterocarpus sp Anogeissus sp Anwa	15	50	...	14.02
AnlofassoSite 4	Anlo fasso	Unknown	30	231	...	-

The values in the “Most abundant species” column indicate the dominant tree species found at each site, with some cells containing multiple species separated by spaces. The “Median Age” column represents the median age of trees at each site.

The “No. of trees per Site” and “Stock Density/m^2^” columns provide information about the tree population at each site. The “No. of trees cut to make charcoal” and “Percentage of tree removal” columns quantify the extent of tree removal for charcoal production.

The remaining columns provide information related to land area, charcoal production metrics (number of bags produced, bags per tree, trees per bag), and land use efficiency (area of land per tree and per bag of charcoal). For the complete dataset, see [2].

It's important to note that some cells in the dataset contain missing values, represented by empty cells or dashes.

## 4 Experimental Design, Materials and Methods

### 4.1 Study area and site selection

The study area focused on the Afram Plains in Ghana, known for its charcoal production activities. Four communities within the Afram Plains were selected for data collection: Tease, Odumasua, Anlo Fasso, and Forifori.

### 4.2 Data collection process

Semi-structured interviews were conducted with community elders, chiefs, and charcoal producers during reconnaissance visits to understand the history and impact of charcoal production on the vegetation of the Afram Plains. These interviews followed a flexible format with predetermined open-ended questions, allowing for exploring topics that emerged during the conversation. The interviews were in-person, typically at the residence or charcoal production sites. They focused on understanding the historical context of charcoal production in the area, local knowledge about the practice, and its perceived impacts on the environment. The semi-structured nature of the interviews allowed participants to share their experiences and insights freely while ensuring that key topics were covered consistently across all interviews. Each interview lasted approximately 45–60 min and was conducted in both English, interjected with the local language where more clarity was required.

With the assistance of charcoal producers, the following data was collected from 29 charcoal production sites:

- Land use of the charcoal production site
- Number of trees per charcoal production
- Number of trees cut to produce charcoal
- Diameter, length, and diameter of tree stumps (for a sample of sites)
- Local names and scientific names (identified with the help of forestry officers) of tree species used for charcoal production
- Number of bags of charcoal produced per mound

Tools and Technology

- GPS fields area measure app: Used to estimate the perimeter of the land area harvested for charcoal production.
- Handheld surveyor's tape: Recorded the length of tree stumps.
- Flexible measuring tape: Measured the diameter of tree trunks.
- GIS cloud mapping software - Mobile Data Collection Portal (https://mdc.giscloud.com/?): A real-time mapping software used to record coordinates of charcoal sites and pre-loaded queries for data collection.
- Mobile app: Data was collected using a mobile app synchronized with the GIS cloud mapping software.

Data Preparation and Analysis

- Data Download and Cleaning: Raw data from the two projects was downloaded and cleaned, assigning unique identifiers and organizing the information into rows (observations) and columns (attributes). Missing values were addressed using field notebook records.
- Attribute Calculation: Various attributes were calculated, including the number of tree species per plot, stock density, and land area demanded for charcoal production.
- Data Exploration and Visualization: Excel and ggplot2 in the tidyverse package of R software were used for initial data exploration and visualization.
- Area Calculation: Perimeter measurements were converted into areas assuming circular shapes using an online circumference to area calculator.

## Limitations

The dataset on urban charcoal consumption and its impact on tree cover loss in Ghana offers valuable insights, but it is important to acknowledge certain limitations. Firstly, the data collection process relied on charcoal producers' recollections and experiences, which may introduce subjectivity and potential underreporting. The estimates of tree ages, for instance, were based on the charcoal producers' observations, which could vary across individuals and be influenced by their specific knowledge and biases. Additionally, the study focused on a limited number of communities in the Afram Plains, which may not fully represent the diverse practices and impacts across the entire region.

Logistical challenges, including access to remote areas and resource constraints, could have impacted the comprehensiveness of the data. The inability to directly measure the diameter at breast height (DBH) of sampled trees due to felling also limited the assessment of tree sizes and growth patterns. The estimation of tree ages, based on spot data collection, may not capture the full variability in age distribution. Furthermore, historical data on charcoal production and tree cover loss in the area was limited, making it challenging to establish long-term trends and patterns.

While the dataset provides valuable insights into the impact of charcoal production on vegetation cover, future research would benefit from expanded data collection efforts, incorporating more diverse communities and addressing logistical constraints. The integration of scientific methods for tree age determination, would enhance the accuracy and reliability of the data. Additionally, long-term monitoring and the collection of historical data could provide a more comprehensive understanding of the dynamics between charcoal production and tree cover loss in Ghana.

## Ethics Statement

The current work does not involve human subjects, animal experiments, or any data collected from social media platforms. The authors have read and followed the ethical requirements for publication in Data in Brief.

## Credit Author Statement

The author is responsible for the whole scholarly work around this article which includes conceptualization, methodology, investigation, writing the original draft.

## Data Availability

Mendeley DataTree Charcoal Data Afram Plains (Original data).
